# C-954-04. Burn Orthoses Clinical Competency Tool

**DOI:** 10.1093/jbcr/irag033.136

**Published:** 2026-04-06

**Authors:** Jennie McGillicuddy, Lynne Benavides, Trudy J Boulter, Andria Martinez, Kerry Mikolaj, Derek O Murray, Nichole Schiffer, Renée Warthman

**Affiliations:** University of California San Diego Health, San Diego, CA; Rhode Island Burn Center, Providence, Rhode Island; Children's Hospital Colorado, Aurora, CO; Diane & Bruce Halle Arizona Burn Center - Valleywise Health, Phoenix, AZ; Children's Hospital Colorado, Aurora, CO; Diane & Bruce Halle Arizona Burn Center - Valleywise Health, Phoenix, AZ; Children's Hospital Colorado, Aurora, CO; Diane & Bruce Halle Arizona Burn Center - Valleywise Health, Phoenix, AZ

## Abstract

**Introduction:**

The use of orthoses, including splints and casts, is an important intervention utilized throughout the continuum of burn rehabilitation. Competencies in healthcare are used to support clinicians by teaching practice standards, establishing clinical expectations for professional development, and by evaluating and improving the effectiveness of educational programs. The authors identified a need to develop a burn-specific pediatric and adult orthoses competency tool for orthoses used throughout each phase of burn rehabilitation.

**Methods:**

A Burn Orthoses Competency was designed by burn therapists from different centers with several advanced credentials working with adults and pediatrics. The competency was designed to align with the Burn Rehabilitation Therapist Competency Tool by Parry et al in 2011 for a tiered training approach. The goal of this competency is to support therapist training while outlining burn orthoses principles and facilitating clinical reasoning with the selection, fabrication, and implementation of orthoses.

**Results:**

The developed competency includes basic principles of burn orthoses for the upper extremity, lower extremity, head and neck, and pediatric considerations, as well as hands-on and documentation sections. The Burn Orthoses Competency is a tool designed to encourage clinical reasoning for burn orthoses selection based on anatomical, cutaneokinematic, and orthoses related principles. It is written in Competency Based Orientation format. Specific components outlined for review include the orthoses name and type, phase of wound healing, positional goals and targeted joints involved, indications and considerations, dosage, application details, and case examples. It is designed to accompany a Burn Orthoses Atlas, being updated by the same authoring group. A reference document, pre-test, and post-test were developed to directly support this competency.

The competency is designed to be a living teaching tool, updated as needed to fit the training needs for each area used. A validator may pull the needed sections based on the role of the therapist being trained, while aligning with previously published burn therapist competencies for tiered training.

**Conclusions:**

The use of current and further development of custom orthoses is impactful in promoting best patient outcomes. Enhancing therapist education on this topic assists to facilitate clinical reasoning with the use of orthoses. Use of tiered training allows for time management of the validator with competency sign off on complex and multidimensional topics, while encouraging use of current literature and resources.

**Applicability of Research to Practice:**

Professional competency should never be viewed as an endpoint, as it goes beyond initial teaching and perfecting skills. The use of a Burn Orthoses Competency allows opportunities for education and professional development while clearly defining standards for best practice.

**Funding for the study:**

N/A.

